# How might the ‘Icelandic model’ for preventing substance use among young people be developed and adapted for use in Scotland? Utilising the consolidated framework for implementation research in a qualitative exploratory study

**DOI:** 10.1186/s12889-021-11828-z

**Published:** 2021-09-25

**Authors:** Hannah Carver, Peter McCulloch, Tessa Parkes

**Affiliations:** 1 Salvation Army Centre for Addiction Services and Research, University of Stirling, Stirling, United Kingdom; 2 School of Health Sciences, University of Dundee, Dundee, United Kingdom

**Keywords:** Adolescence, Prevention, Substance use, Icelandic model, Qualitative, Scotland

## Abstract

**Background:**

Substance use among young people is a significant public health concern, particularly in Scotland. Primary prevention activities are essential in delaying young people’s substance use and reducing the harms associated with use. However, such prevention activities are generally lacking. The Icelandic Model (IM) has received increasing attention and has been associated with improvements in substance use in Iceland since the 1990s. There is interest in implementing the IM in Scotland but concerns regarding transferability. This research study aimed to address a gap in the evidence base by providing insight into stakeholders’ views of the IM in Dundee and more widely in Scotland.

**Methods:**

Qualitative data were collected via semi-structured telephone interviews with 16 stakeholders. Data were analysed using Framework Analysis in NVivo, informed by the Consolidated Framework for Implementation Research.

**Results:**

Participants were keen for more prevention activities to be delivered in Scotland and were generally supportive of the IM, given the high rates of substance use and related harm. A range of positive factors were identified, including the evidence base, the multi-component nature of the IM, and availability of current services that could be embedded into delivery. Several barriers were noted, relating to funding, the franchise model, support and buy-in and cultural differences.

**Conclusions:**

Our findings provide insight into the views of a range of stakeholders regarding the potential implementation of the IM in Scotland, and perceived barriers and facilitators. There is a desire for primary prevention activities in Scotland, driven by concerns about high rates of substance use and related harms, and a general lack of effective and evidence based prevention activities across the country. Several key barriers would need to be addressed in order for implementation to be successful, and participants were clear that initial piloting is required. Future research and evaluation is required to examine its potential and the outcomes of the approach in Scotland.

**Supplementary Information:**

The online version contains supplementary material available at 10.1186/s12889-021-11828-z.

## Background

Globally, most young people begin experimenting with alcohol, tobacco and drug use during adolescence [1–3]. Typically, alcohol use begins between the ages of 14–21 years, tobacco between 15 and 21 years and drug use between 16 and 28 years [4]. In Scotland, substance use amongst young people is a significant public health concern, with many reporting alcohol, tobacco and/or drug use [5, 6]. In the most recent Scottish Schools Adolescent Lifestyle and Substance Use Survey (SALSUS), 20% of 15 year olds reported using alcohol in the last week, 12% used drugs in the last month and 7% were regular smokers [6]. While rates of substance use among young people in Scotland has reduced over the last decade, and rates of smoking remain unchanged since 2015, there have recently been increases in alcohol and drug use [6], reflecting a different pattern compared to across Europe [7]. Young people in Scotland report starting to use substances at an early age, at between 13 and 14 years across all substances [6]. The earlier a young person uses substances, the more likely they are to use them more frequently and develop problems [1, 2, 8, 9]. There is increasing evidence of the negative impact of substance use on young people’s health and wellbeing, and the impact on the key transitions experienced during this time [4]. Therefore, primary prevention of adolescent substance use is a key public health priority.

Primary prevention activities are essential in delaying young people’s substance use until they are older and reducing the harms associated with use if they start using substances. Such activities are divided into universal, selective and indicated interventions [10]. Universal interventions focus on all young people, irrespective of their risk of substance use; selective interventions focus on more vulnerable groups; and indicated interventions focus on individuals to reduce their risk of developing problems with substances [10]. Prevention activities can include restricting access to substances (such as through minimum legal age); taxation; banning advertising of products; mass media campaigns; school based education; and family based interventions [11]. These interventions aim to deter young people from using substances in the first place. Despite a wide range of interventions available, the evidence base is somewhat limited compared to other interventions. Relatedly, prevention activities receive far less funding than other interventions such as treatment. There is some evidence of the effectiveness of some school and family-based interventions [12–15]. In Scotland, the delivery of Alcohol Brief Interventions has been broadened to include wider settings, including youth services, as part of the Scottish Government’s national programme, to address high rates of alcohol use [16]. Additionally, the current Scottish Government drug and alcohol strategy [17] notes the need for new universal prevention/education approaches for young people. While steps have been taken to increase provision, substance use prevention activities are somewhat lacking in Scotland, despite a focus on prevention [18].

In recent years there has been increasing attention given to Iceland and the country’s approach to prevention. Iceland had problems with adolescent substance use during the 1990s, with rates higher than many countries [19, 20]. In 1998, 42% of 14–16 year olds reported being drunk in the last month, 23% being daily smokers, and 17% had ever used cannabis [20]. Since implementation of a new approach, the Icelandic Model (IM), rates of alcohol, tobacco and drug use have decreased dramatically [20], with drunkenness rates reducing to 20%, smoking to 10% and cannabis use to 7% in 2007. The rates of use have continued to reduce over the years, with even lower rates of substance use now reported [21]. Along with these reductions in substance use, there is evidence of increased protective factors, such as spending more time at home and participation in organised sports [22, 23] A recent study has shown that the key features of the IM are likely to have a positive effect on substance use outcomes [24]. The IM is a universal, community-based approach aiming to prevent young people’s substance use through reducing risk factors and increasing protective factors [20]. Key components are: parents, organised extracurricular/recreational activities, schools, and the involvement of young people [25]. Schools are urged to strengthen supportive networks with parents, and other community groups [25]. Activities also include: subsidised recreational/sporting activities; encouragement of parental monitoring and family dinners; curfews; strong alcohol policies, particularly in relation to minimum ages and advertising; and improved social norms [20, 26]. A thorough annual survey is provided to 14–16 year olds to understand substance use and risk and protective factors, which then informs the interventions that can be implemented [20, 26]. The approach has been implemented in more than 30 countries worldwide, with adaptations to suit locally specific conditions [27, 28]. Planet Youth, a team of academics based at Reykjavik University, are responsible for the delivery of the IM, with organisations paying to become part of the franchise [29].

Despite the growing popularity and interest in the IM worldwide, there are concerns regarding the transferability of the IM to other countries and cultures [26, 30]. Currently, almost all of the evidence regarding programme effectiveness comes from Iceland, despite the IM being implemented in many countries worldwide, with one recent study showing outcomes from Lithuania [31] and planned research in Canada [32]. Relatedly, there is limited definitive scientific evidence regarding the effect of IM on young people’s substance use. While the Planet Youth team report dramatic reductions in substance use over the last 20 years, such reductions have also been observed elsewhere in Europe [26]. Koning et al. (2020) urge those considering implementing the IM to critically review the evidence base, particularly in terms of cultural and contextual differences [30]. Thus, more research is required to understand the implementation of the IM in other countries. In Scotland, there is increasing interest in implementing the IM. The aim of this study was to understand whether the IM should be implemented in Scotland, with particular focus on one city, Dundee. This study is linked to a wider knowledge exchange study funded by the Society for the Study of Addiction to convene a co-production process involving a diverse group of individuals living and working in Dundee to review and interrogate the evidence base of the IM and provide recommendations regarding its implementation in the city (for more information see https://www.addiction-ssa.org/yiim/). This research study aimed to address a gap in the evidence base by providing insight into stakeholders’ views of the IM in Dundee and more widely in Scotland.

The study was informed by the Consolidated Framework for Implementation Research (CFIR [33];). The CFIR provides a comprehensive list of domains and associated constructs thought to influence the implementation of complex interventions [33]. The five domains are intervention characteristics; outer setting; inner setting; characteristics of individuals; and process, with each encompassing a range of constructs [33], which are detailed below. The constructs include the adaptability, complexity and cost of the intervention (intervention characteristics); patient needs and resources and external policies (outer setting); culture and implementation climate (inner setting); knowledge and beliefs about the intervention (characteristics of individuals); and planning, engaging and executing (process) [33]. The CFIR has been used in a wide range of studies within the field of healthcare and most aim to understand practitioners’ experiences of barriers and facilitators to implementation [34]. The framework can be used prior to an intervention being implemented, during and after. It is important to note that, while there are five domains and various constructs of the CFIR, not all will be relevant in all studies [34]. In the current study, the CFIR was chosen as it provides a framework for understanding the most important factors to be considered when implementing the IM.

## Methods

A qualitative study was conducted involving interviews with a range of stakeholders to gather their views of the IM and whether it should be implemented in Dundee and in Scotland more widely. Stakeholders were identified through the research team’s networks in order to identify relevant individuals working in the field of substance use prevention. This included national organisations, third sector organisations, statutory agencies, and family members with lived experience of substance use. The research team created a list of individuals and organisations who were known to be interested in the IM approach, as well as individuals who have a remit for prevention activities, such as those in Alcohol and Drug Partnerships (ADPs). In Scotland, ADPs are responsible for commissioning substance use services in local areas [35]. To gather a range of views, we contacted two groups of individuals: those who had expressed interest in the approach, in that they had been in touch about the wider project, had attended relevant events/conferences, were part of the wider project team, or had expressed an interest more informally; and people we had not been in touch with who may be interested or involved in substance use prevention activities. This provided an initial list of 30 individuals from ADPs, national organisations, statutory agencies, third sector agencies, academia and family members. Purposive sampling was used to select individuals based on their interest in the approach, the group they represented and the area in which they are located (Dundee, another area or Scotland wide). If participants were unable to participate, they were asked to suggest a relevant colleague. Participants were also asked to suggest people that they thought might be less familiar with or less favourable towards the IM, in order to gather a wide range of views.

Semi-structured interviews were conducted by one researcher (PMcC) during May and June 2020. Individuals were contacted by email and invited to participate in a telephone interview. Written informed consent was granted prior to each interview. The interviews were all conducted by phone, were audio recorded, and lasted an average of 53 min. The interview schedules covered: views on current prevention activities; the role of different stakeholders in prevention; the IM and barriers and facilitators to implementing the approach in Dundee/Scotland and differed slightly for Dundee and national participants (Additional File 1). After each interview, participants were provided with a debrief sheet (to provide further information about the study and support available). Detailed fieldnotes captured researchers’ experiences and reflections of the interview as a way of enhancing reflexivity [36]. These also supported small changes to the interview schedule.

Data were transcribed in full and analysed using Framework Analysis [37] in NVivo 12. Framework Analysis is suited to policy-relevant research and provides a structured and transparent method of data analysis [38]. Following the stages of Framework Analysis, the transcripts were combined into one dataset and read in full, then coded line by line in NVivo by PMcC. Coding was inductive to allow new ideas to emerge, as well as being guided by the research questions. After coding the first four transcripts, the initial thematic framework was developed by PMcC and checked by HC; this was then used to code the remainder of the transcripts. Participants were provided with pseudonyms. Ethical approval for the study was granted by University of Stirling’s General University Ethics Panel (GUEP; paper 859).

As noted above, this study was informed by the CFIR, which provided a way of understanding the factors that might influence the implementation of the IM. We adopted a post-hoc application of the CFIR. Once the initial themes had been identified, relevant theoretical frameworks (including but not restricted to the CFIR), were cross checked with the themes to identify best fit. It was determined that the CFIR was best suited, as all themes could be allocated conceptually within four of its associated domains and constructs, to provide a greater theoretical understanding of the findings. The data were then sorted and re-arranged into themes and sub-themes, corresponding to the domains and constructs of the CFIR, with quotes chosen to illustrate key points (by HC and PMcC).

## Results

A total of 16 interviews were conducted. Table 1 provides interview participant characteristics, in terms of their current job role. Family members were those who had a child with experience of problem substance use. Of the 16 participants, eight were from Dundee and the others from Scotland more widely.

**Table 1:** Interview participant characteristics

Government	*N* = 2
Health	*N* = 2
Third sector	*N* = 5
Family member	*N* = 4
Local authority	*N* = 2
Academia	*N* = 1

The data fitted into four of the five domains of the CFIR: intervention characteristics, inner setting, characteristics of individuals, and process, in some but not all of the constructs. The domain ‘outer setting’, which relates to the external features that may be pertinent to implementation, was not applicable to the data collected. In this study, Scotland was defined as the implementing organisation, rather than a discrete organisation within Scotland, making traditional external influences unlikely to emerge. Furthermore, as noted above, is not necessary for all domains and constructs to be included when using the CFIR [34]. Table 2 below details the domains and constructs of the CFIR. *Those deemed not relevant to this study have been highlighted in grey*. The findings are described in terms of the four domains, divided into key themes (which are mapped onto the key constructs), with illustrative quotes from participants. Some themes encompass multiple constructs and are identified in brackets at the start of each theme.

**Table 2:** CFIR domains and constructs

Intervention characteristics	Inner setting	*Outer setting*	Characteristics of individuals	Process
•-Intervention source •-Evidence strength and quality •-Relative advantage •-Adaptability •-Trialability •-Complexity •-Design quality and packaging •-Cost	*•-Structural characteristics* •-Networks and communications •-Culture •-Implementation climate •-Readiness for implementation	•*-Patient needs and resources* *•-Cosmopolitanism* *•-Peer pressure* *•-External policy and incentives*	•-Knowledge and beliefs about the intervention *•-Self-efficacy* •-Individual stage of change •*-Individual identification with the organisation* •*-Other personal attributes*	•-Planning •-Engaging *•-Executing* *•-Reflecting and evaluating*

### Intervention characteristics

Intervention characteristics refers to the key components of an intervention that are thought to influence successful implementation [33]. In the study, this related to perceptions of the IM (intervention source, evidence strength and quality; relative advantage; complexity); adaptability of the IM (adaptability); the need for piloting (trialability); and the Planet Youth franchise (design quality and packaging; cost).

#### Perceptions of the IM

Some participants discussed the lack of prevention activities in Scotland, which provided justification for the need for an alternative approach. There was a view that current rates of substance use in Scotland are problematic, and that something needs to be done. The IM was perceived as having the potential to reduce high rates of substance use across the country:

We have a major problem and it has to be tackled. We cannae [can’t] keep going on generation after generation with people with alcohol and drug, mental health issues. Because it’s huge, mental health issues, and we can’t carry on like that… [without the IM] I think you would get much more people that were dependent on alcohol and drugs in Dundee and you’d get much more drug deaths. I think we’ve got to tackle it. (Becky, family member)

Participants described the importance of the implementation of the IM being driven by both a top down (strategic) and a bottom up (community driven) approach. This reflected an understanding that the IM will not be committed to by stakeholder organisations if it is not prioritised, and will not gain momentum or success without community buy-in. While buy-in might take more time to gain, having community stakeholders on-board could convince strategic partners of the importance of the IM, as described by Stuart (third sector):

There would need to be a little bit more work done potentially to get people on side in Dundee. That might just take a little bit more time… that would just be a little bit more maybe how this would be marketed and how it would actually be sold to people to actually let them see and understand what it is and what it’s trying to do.

Awareness of the IM and the breadth of the evidence base appeared to facilitate buy-in to the possibility of implementation in Scotland, with participants noting that the evidence base for the IM appeared to be stronger than for other prevention approaches:

I’m seeing this [IM] as something that is coming with quite a sound evidence base… seems to come with a much stronger evidence base than anything I’ve been aware of before … I think it brings a credibility… if we are going to be making a pitch. (Christine, local authority)

The benefits of the IM are the multiple components and stakeholders. However, these elements also add to the complexity of implementation. Participants discussed the challenge posed by the volume of tasks required for implementation, including high rates of substance use, large local authority areas, partnership working, prioritisation of the approach, and the involvement of numerous stakeholders. However, Rose (health) noted that the complexity of the IM is “both its beauty and also it’s [a] huge, huge challenge… it is just massive".

#### Adaptability of the IM components

Most of the major components of the IM were viewed as important factors to encourage prevention of substance use among young people in Scotland. Participants discussed these components in relation to current provision in Scotland. In terms of the surveys, there was recognition that there is a lack of data relating to young people’s substance use in Scotland, which Susan (third sector) described as “really quite serious”. The IM surveys were viewed as a beneficial way to gather data from young people in order to identify issues and target interventions, in an “evidence based” (Stuart, third sector) and “scientific” (Deborah, family member) manner. Current surveys in Scotland (SALSUS) provide local authority level data, but the IM survey was perceived to be superior, by providing more localised, school-based information:

The localised information is so, so important. The fact that you can point towards the fact of these are responses from the kids at this school, or this local area, whatever it might be. And it’s applicable to a community. I think that’s hugely important. So the outcomes are, if you can go back to a school, a Director of Education or a local council or an MSP whoever it might be and say this isn’t overall stats for the whole of Scotland, or for a whole local authority. This is the stats for your local schools, or the local kids in your school, and then it becomes a lot more powerful… you know there is no hiding place in that one. (John, third sector)

This localised information was viewed as being beneficial in holding decision makers accountable, to convince them of the need for the IM and associated interventions. There was, however, awareness of the challenges of conducting surveys with young people and being able to get “real honest answers” (Christine, local authority), due to concerns about confidentiality and data protection. Concerns were also raised regarding the involvement of more marginalised young people, especially if they are disengaged from school, as described by Susan (third sector):

That most important population of young people who don’t engage in school, we know that they have the highest risk probably in terms of drug harms, so I don’t know why we are basing policy on a survey that doesn’t, you know, it just doesn’t reach them.

As in Iceland, schools were perceived as having a key role in IM implementation in Scotland. Participants viewed schools as being able to provide activities, with buy-in from staff being essential in terms of funding allocation and involvement in the survey. Peer influence was also viewed as an important component of the IM. Susan (third sector) noted the positives of peers and the “sustaining and nourishing work that they have in terms of their friendship”, viewing young people’s friendships as something to be supported. These positive peer roles were discussed in terms of peer education models (for example in delivering the survey) and the importance of youth clubs, and as something to be considered for implementation.

Recreational activities were discussed by several participants as a positive part of the IM and essential for substance use prevention, although some noted the current lack of funding for these activities in Scotland. Such activities were viewed as diversionary, providing something for young people to do instead of using substances, and as a way of relieving boredom. Participants described the range of activities already on offer in Scotland stating that implementation of the IM should “capitalise” (Sarah, local authority) on these activities, ensuring adequate funding, sustainability and improved links with schools. Involving a range of stakeholders in the delivery of these activities was viewed positively, by increasing provision of activities that are already provided, as noted by Christine (local authority):

… lots of different pieces to the jigsaw here that could come together to provide a whole package of offer. There is great opportunity here. There is a lot of work to be done to pull it together but I still think you know we can join up other agenda’s. It’s about piggy backing on things that are already happening, and growing it.

Ensuring access to these activities was viewed as important, for example by supporting more marginalised young people to engage, and also through providing travel expenses or travel options to take young people to and from activities. Becky (family member) also noted the importance of funding to provide transport for young people to get to sports and recreational activities, as it is likely that without such transport, those from deprived areas would be less likely to attend.

Parents were also seen as key in IM implementation and in prevention more generally. Current involvement of parents in prevention activities in Scotland was viewed as somewhat lacking. Participants acknowledged that involving them may be challenging, particularly those who are more marginalised:

It would take quite a bit of persuasion to get parents involved. There is a huge amount of just get the kids away some place so that they can get time on their own. A lot of them it’s … affordability [of sports/activities]. I think it could work but I think it would take a lot of work to get it working, at the start. (Fiona, family member)

Steve (third sector) also noted potential challenges around families being mistrusting of particular services, including the Police, which could impact upon their engagement with the IM. Participants suggested ways of engaging with parents, including communicating with them directly, instead of through their children, through technology and social media, and meetings with parents, as well as asking parents to commit to the IM by signing a pledge.

#### Piloting the IM is essential

Participants discussed the need for the IM to be piloted in Scotland before wider implementation, due to concerns about the validity of findings, the complexity of adapting services in Scotland to suit the model, the need to determine whether the IM is worth investing in, and to increase stakeholder buy-in. Piloting would provide much needed evidence to determine whether it is worth investing in the model and rolling it out nationally:

You would have a long time before you would have any results to know whether you wanted to throw your money at it. But if the government were really serious then they should be supporting like a couple of pilot sites, or three pilot sites, or something. There would be real value to that. My question really would be to the Scottish Government, why are they not doing it? (Ashley, health)

Pamela (third sector) also noted the importance of involving young people in the process of piloting and wider implementation, for example through discussing the IM with policymakers in terms of the benefits on them and their communities:

If we have a pilot in Dundee, where this will actually give young people what they wanted. Give them a sense of empowerment and self-esteem and achievement. If those young people could speak to the politicians themselves that would be powerful… if families could talk to government and elected members locally and MSPs about the difference this could make for their children and young people, particularly if they have got parents who you know, a lot of families affected by drugs… I think they could make a very compelling case.

#### The Planet Youth franchise

For some participants, the established franchise of the IM provided a level of credibility in terms of the evidence base. A perceived advantage of buying into the franchise model provided by Planet Youth was that their experience across the world may facilitate a smooth and credible implementation in Scotland which could be used promote and encourage stakeholders and decision makers to engage:

It’s much easier to sell something that’s been packaged and marketed really well. And it’s got that international backing. Like there is a value to that… it’s a really exciting opportunity. For me I think it’s, I think I’ve said this already, but these aren’t new ideas, but what it does do is provide us a greater… a stronger case in a clearer package for how this could be used. And I think it would be easier to sell if it comes like that. (Ashley, health)

Relatedly, some participants were sceptical about the Planet Youth commercial endeavour arguing that local expertise in Scotland could develop something similar for far less money. Alex (academic) described their concerns:

They’ve commercialised it… what they are doing actually is selling a model which is a general model that is out there for anybody to use and build on… What they are actually selling is their work to evaluate and provide data regarding what’s happening, you know, when you implement the model… I don’t know, I might be prejudiced it’s just when I see something that is being totally PR led in my view I tend to not like it… I can’t see the point you know of just paying, unless you are doing exactly the same as they do. Why pay their people to do your, you know your outcomes and measure your outcomes… Why don’t you get a Scottish University to say, these are the appropriate measures, this is what we are looking for? Why do you pay a commercial… what is now a commercial organisation actually to do something that really, in my view, could be done in-house?

Ashley (health), however, noted that there would be issues around developing an independent Scottish model and was unsure as to whether this would be more beneficial than buying into the existing Planet Youth model.

### Inner setting

Inner setting refers to the structural, political, and social contexts related to the implementation of the intervention [33]. In the study this related to current prevention service provision in Scotland (networks and communication); the need for new interventions (implementation climate); cultural differences between Scotland and Iceland (culture); support and buy-in (implementation climate); a potentially resistant political system (implementation climate); competing priorities (implementation climate); availability of funding (readiness for implementation); and awareness of/knowledge about the IM (readiness for implementation).

#### Current prevention service provision in Scotland

Most participants perceived that youth prevention services were somewhat lacking in Scotland, with more focus on frontline addiction services for adults. Deborah (family member) reflected on their experience as a parent and felt that much of the prevention work centred on education in schools which typically followed a ‘just say no’ approach, an approach that she felt was unrealistic for many:

I’ve looked online, I don’t know what prevention programmes there are in Dundee. There is nothing jumping out at me. I am not saying it’s not there, but I can’t see anything that talks about prevention. So if I was to think about the schools, I am fairly sure they do have some prevention programme. However, prevention is not going to happen if you say to kids, just say no. We know that doesn’t work.

Other participants were aware of some good prevention work in Scotland and felt that utilising and developing good partnerships with existing services was key in implementing the IM in Scotland. This appeared to be particularly important in response to the COVID-19 pandemic, which had resulted in reductions in resources, services and staff:

The key is to get to grips with the fact that we have got such limited resources at the moment and it’s going to be even more stretched post COVID-19. So we will be seeing cuts to services in a frightening way I would imagine. So the key will be to make best use of what we’ve already got and align it to the things that we know make the difference… We just did a huge engagement process between November and the end of March with the public and people with lived experience. People felt there was a greater need for more positive alternative activities for people to do other than using alcohol and drugs. That is clearly a kind of a linchpin of the Icelandic Model, it’s all about those activities and those opportunities. So one of the keys for it is you don’t set up a new thing where you provide activities for young people, you have to properly understand what is already available in an area... And then work out where the gaps are, and also work out why young people are not accessing the stuff that is already there, if they are not. (Ashley, health).

#### The need for new interventions in Scotland

It was suggested that the lack of prevention activities in Scotland contributed to the high rates of alcohol and drug use and related harm. Drug and alcohol use were seen as culturally acceptable activities in Scotland, leading to high rates of use:

You’ve got other people, I’m just talking about like mainstream, maybe your sixteen to twenty year old, you are getting into cocaine as a normal thing. It’s kind of becoming part of growing up… then you’ve got, on the other hand, there is the heroin and the Diazepam use. Now a lot of young people that are finding that and getting involved in… then that’s kind of become the norm… it’s just a lot more available. It’s easy money and it’s also an easy way to escape from their surroundings (Steve, third sector)

There was a sense that new interventions like the IM are needed in Scotland to address high rates of substance use and related harms by following an evidence based approach. Participants talked about the need to do things ‘differently’, and implementing, or at the very least piloting, the IM was suggested as a way forward:

This [IM] is something we should really be doing because we haven’t really given young people what we need to. We need to really improve what has been there [previous interventions]. We have learnt that we’ve got the most awful drug related deaths in Scotland, we have to do things differently. (Susan, third sector)

#### Cultural differences between Scotland and Iceland

Iceland is typically seen as different to Scotland in terms of its normative culture, use of substances, schooling system, provision of services, and welfare provision [26, 30]. This perception led participants to reflect on the need for the IM to be adapted for use in Scotland:

Certainly, there is nothing to suggest that any model, not just this one, can just be taken from one cultural specific context and implemented in the same way in another. (Alex, academic)

Deborah (family member), however, noted that, while Scotland and Iceland differ culturally, the IM has been implemented in many countries worldwide with apparent success, providing evidence for its potential success in Scotland. However, the unsuitability of the curfew as an intervention within the IM was identified as one element with limited transferability:

You have to look at each of these elements and I think there is fairly general agreement that some of them wouldn’t work in Dundee. You know, the curfew for example… You’ve got to look at these elements which make a multicomponent whole and say ‘no we can’t do that in Scotland, or in this city, or in this context’, wherever it happens to be. We have to look at our context and see what can we do, what is possible, what kind of multicomponent model can we build that suits our circumstances? (Alex, academic)

Despite these concerns, there was a view from some that the curfew could in fact be implemented, in a slightly revised format. For example, Deborah (family member) suggested that a more informal approach, an agreement between parents and their children, would be much more appropriate, and reflect Scottish culture:

Well my parents had a curfew. I had to be in by a certain time. But I had a curfew, my parents did it, it wasn’t in law. So I think if you adopt the Icelandic Model in it’s vast majority of actions, you can, if there is a will, replicate. Those kids in Iceland aren’t any different from our kids. (Deborah, family member)

#### Support and buy-in for the IM

Participants were generally supportive of the IM and the possible impact it might have on reducing substance use among young people. While participants were generally supportive of the IM and potential implementation in Scotland, they reflected on the extent to which there was buy-in across the country. For example, John (third sector) and Sarah (local authority) noted that engagement with the IM was at an early stage and that, while there is a willingness to adopting the approach, no concrete steps have been taken to implement it, in terms of practical or financial investment. Sarah noted the extent of buy-in within the education department:

Some colleagues are very, very keen on it, I am aware of that. I am also aware that some key people within the education department are very keen on it… How much thought they have actually given to the implications of it, and what would it actually mean in practice and all of that, I am not sure. I think we still have some discussions to do. But my sense is that there is… yeah, there is willingness.

Increasing buy-in and ownership across all potential stakeholders was perceived as an important factor in the likelihood of implementing the IM approach in Scotland.

#### A potentially resistant political system

Several participants mentioned their concern that the political set up in Scotland may not be ideal to foster support from politicians. It was suggested that key decision makers may not buy-in to the model due to the focus on short term outcomes in relation to election cycles. There was also active resistance to discuss the IM from some local councils, as noted by Craig (family member). Ashley (health) sums this up clearly:

But my feeling is that it’s always been quite hard to make the case for prevention because it is so slow for you to see a difference. Politically not a great you know not a popular choice, and then also the other things is that you are always under a lot of pressure from people like elected members who think that education in schools is the answer. So they say, oh yes, if we just educate young people about the dangers of alcohol and drugs then everything will be fine. And, of course, it’s absolute nonsense.

Cross-party support may therefore be required to overcome these barriers. This would ensure that prevention activities, including the IM, would continue to be supported and funded, regardless of which political party were in power. William (government) noted that such an approach would ensure that benefits could be seen in the long term:

It would need to be, in my opinion, run for a long time if it was going to work, and there would need to be a cross political party commitment to keep it going for a number of years I would say, to be honest… if they took a longer-term view they would see that in the long run it would actually reduce costs to the NHS and councils and social work and the police for that matter.

#### Competing priorities

Participants talked about the challenges of competing priorities within and between organisations as a barrier to the successful implementation of the IM in Scotland. For example, organisations are working towards particular goals, are committed to many projects, and staff are overworked. Their view was that the lack of priority placed on prevention work overall in Scotland also played a part, with limited strategic buy-in regarding the need for, and delivery of, prevention activities. Again, the focus on drug related deaths was seen as being prioritised over prevention work:

We are still in the position where we are reactively responding to the pressures that are arising and, again, I just don’t think that child health substance use risk, children with substance use, is high enough in the Alcohol and Drugs Partnership communications in terms of, as I say, the Commission Report, Taskforce recommendations. I’m not sure how prominent it is in respect to recovery. (Rose, health)

The development of an approach which facilitated collective responsibility for prevention activities was suggested as a way of overcoming this barrier. Such an approach was seen as ensuring prevention activities could be prioritised. Pamela (third sector) talked about the success of countries who had implemented legal requirements for prevention work to be delivered in local authority areas, noting that making investments in prevention resulted in “better outcomes for young people but also for the wider community, the economy and so on”.

#### Availability of funding

As discussed above, the lack of focus on prevention activities in Scotland was discussed as a key concern and barrier in the implementation of the IM approach. Related to this was the view that there is a lack of funding for prevention activities across the country, and that there are also inconsistencies in levels of funding within/across different local authority areas. Sarah (local authority) noted that partnership working between several third sector services may be a way of overcoming these funding challenges. Pamela (third sector) noted the need for these third sector services to receive adequate long-term and holistic funding (rather than short-term funding of specific services/activities) in order for services and activities to be delivered successfully. Such funding would allow more flexibility in what can be offered and to whom:

We need to think of a radical shift in the way that funding is allocated… because of the way that funding works and because of the scarcity of funding… You then have organisations that are delivering work to fit funding reports rather than delivering work that the community needs… I can think back to a few organisations who got funding to do a specific thing but then, by the time the funding was awarded, we’ve got the young people together, no we are not interested in that, we want to do this and this instead, but I still had to go on with the project… rather than completely changing what we were doing and be able to spend that money freely and go ‘well actually ten times the number of young people will be engaged with this other project, so let’s do that’.

There was recognition that the COVID-19 pandemic had reduced the amount of available funding, potentially making the implementation of the IM less likely. Participants suggested other means of gaining funding such as private businesses investing in prevention activities. Participants also discussed the ethical dilemma of whether alcohol or tobacco industry funding could be accepted. John (third sector) was sceptical about the benevolent offers of funding from the industry, while Susan (third sector) noted the potential of such funding to reduce the harm caused by these industries. Pamela (third sector) described an approach to funding used in other countries where money from the gambling industry is used to fund prevention activities, and in the USA where taxation from the cannabis industry is used to fund youth activities. However, they appreciated the controversy of such funding.

#### Awareness and knowledge about the IM

Participants were asked about how they became aware of the IM approach. Most were introduced through friends or colleagues and the stories of success from Iceland in the media. Many interviewees described having good knowledge about the IM and appeared interested in the positive impact it could have. However, there was recognition that the approach was not generally widely known, and therefore current support for the IM was driven by a minority of interested individuals. This led some participants to suggest that the IM needed to be promoted more widely, rather than relying on invested individuals disseminating information about it, and prevention more generally. Getting the message out, and sharing success stories of those who have implemented the IM, was seen as critical:

What is really important is to educate people about what is effective prevention and then you’ve got to sort of sell that as a concept. So I think that’s another really helpful thing that something like Planet Youth can bring because there is the power of the story of the people who have been doing this for a long time. (Ashley, health)

### Characteristics of individuals

Characteristics of individuals refers to the influences that people have on intervention implementation [33]. This theme relates to the constructs which may be a barrier or facilitator to the potential implementation of the IM in Scotland more generally: knowledge of the IM (knowledge and beliefs about the intervention) and general buy-in and support for the model (individual stage of change).

#### Knowledge of the IM

As noted above, most participants had a good understanding of the model. When asked about what they knew, they mentioned the key components and their awareness of the reductions in young people’s substance use over time in Iceland. Nevertheless, one participant talked about his apprehension about rolling out the model in Scotland due to a concern that doing so would be an attempt to legalise drug use. This view appeared to come from a lack of knowledge about the IM; when asked about what he knew of the model, William (government) stated: “Not very much to be blunt, you know I have read a bit on it”. Craig (family member) reflected that resistance from senior decision makers may also relate to a lack of knowledge about the model. Data suggests that some stakeholders may need greater understanding of the IM in order to allay fears and encourage engagement, support, and a willingness to shape its potential implementation in Scotland.

#### General buy-in and support for the IM

Participants generally appeared to be in support of the IM and the possible impact it might have on reducing substance use among young people in Scotland. One participant was very supportive of the model, which appeared to be reinforced by the fact that their understanding of the benefit of its components was framed within their area of expertise. For Christine (local authority) the diversionary activities fitted well within the education department’s curricular framework, which contains underlying principles for better outcomes for young people [39]:

Obviously the Icelandic Model… a lot around sport and getting young people involved in what we would have called long ago extra-curricular activities, and we tend to describe that now as wider opportunities in the curriculum. So we are interested in that because it sits very much with our understanding in Scotland around what the Curriculum for Excellence was supposed to be about, not just the core curricular subject area. It was around wider opportunities. And whether these were in sports or recreational activities. But it was about… a young person’s opportunities to learn and grow. So for me this model would sit really well in there.

When asked about other views of the IM, participants reported that they were not aware of anyone who was fundamentally opposed to the implementation of the approach in Scotland. Despite a general feeling of support for the model among the sample, a few participants suggested that willingness to implement the model in Scotland may be in its early stages in terms of the concept, rather than the practical steps towards implementation. Participants talked about colleagues being keen to implement the model but had not yet taken steps to take it forward, for example in terms of identifying funding, areas/schools or stakeholders:

As an organisation we are certainly exploring the fact of looking at pilots in Scotland, potentially. However, that needs all the stuff we’ve been talking about convening, and identifying local authorities, the funding, absolutely. So yes we’ve got the buy in from a purpose level. We’ve certainly not got the buy in from a financial level because we’ve not taken it to that stage yet. But we haven’t made a financial commitment to it in any way whatsoever, we are still exploring it. But as a concept for Scotland, yes we have an invested interest in it. (John, third sector)

### Process

Process refers to the method of change required for the successful implementation of an intervention [33]. In this study, it related to strategic planning and partnership working (planning); key leadership (engaging); and the voices of young people (engaging).

#### Strategic planning and partnership working

Participants talked about the importance of partnership working between multiple stakeholders in implementing the IM, as it relies on a multi-agency approach, which cannot be done in isolation. Such stakeholders included third sector organisations, schools, communities, and parents. Bringing these partners together in order to successfully implement the IM was perceived as challenging. Rose (health) noted the wide range of partners who would need to be involved:

You are talking about Public Health, Education, Council, Housing, and employment. You are talking about Health, about what support there is in schools. Even kind of city planning and outdoor spaces, and parks and diversions. You are talking about community, you are talking about families that people grow up in and their extended family support. You are talking about youth groups, the third sector, voluntary organisations… It’s everything.

Thus, a key step forward in implementing the IM in Scotland would be to consult with a range of stakeholders in order to start taking the process forward if it was decided that the IM should be implemented. Rather than developing new partnerships to implement the IM, there was a sense that effective partnerships already exist between a range of organisations and could be further developed. Participants talked about partnerships within statutory organisations and between third sector and statutory organisations, including NHS and schools.

#### Key leadership

In terms of driving the IM forward, participants had different ideas of who the key leaders should be, and these would likely differ in different areas of the country. For some, the local ADPs were seen as key leaders given their role in commissioning substance use services. However, there was also a sense that the overwhelming focus of some ADPs was on treatment rather than prevention, limiting their ability (or willingness) to take forward the IM:

If you do it in ADPs it gets lost with the treatment side of things… it might be different in other ADPs, but the agenda for ADPs is very treatment focused. It’s all about drug deaths, it’s all about waiting times, it’s all about addiction services. The people that tend to manage them are often addiction services people. There are lots of areas that are doing really good prevention work, don’t get me wrong, but I don’t know if it’s actually the ADP that is driving that. (Ashley, health)

Christine (local authority) felt that education departments may be most suited to leading the IM given the central role of schools within the model:

It seems to me that there is quite an emphasis on what happens in schools in the programme, as I understand it from Iceland, so yes, I am thinking that universal prevention and education bit has got to sit somewhere with us”.

Steve (third sector) described the importance of having good role models and that having people with the “right personalities that can just be a driving force… getting these right personalities involved will definitely be a good thing”.

#### The voices of young people

Participants felt that young people are not often heard from, or included in decision making, with assumptions often made regarding what will be of benefit for them, without asking them about it:

There is a lack of credibility sometimes… I don’t know that young people always take what we offer as credible. The key thing for us to understand is what is it that young people would want to do. What is it that they would actually come to, or would want to take part in? And also how do we make sure that they have a sense of ownership. (Christine, local authority)

Thus, involving young people in the development of the IM in Scotland was viewed as crucial, taking a ‘bottom up’ approach to ensure that the way in which the IM is implemented fits with the needs and wants of young people. This appeared to be particularly important when developing services or activities to address risk factors:

A really key element in terms of establishing the project or, you know, the model is to have an understanding of what is it that would capture the imagination of young people, that they would really want to be part of. Because I think if we succeed in doing that then they would participate, they would take part. (Sarah, local authority)

Participants believed that young people should be involved in the process from the beginning given they are the experts in what is needed for themselves, their peers and their communities. As Becky (family member) explains, listening to young people about the issues they face will enable a better understanding of how to respond to these issues:

Focusing on what children and young people are saying the issues are. I think we need to make sure that we get the, they get the right help when they ask for it. We should identify what the issues are much earlier on and have the resources there at hand to be able to deliver on when it’s needed, not three years down the road.

## Discussion

This study aimed to provide insight into stakeholders’ views of the IM and the potential for implementation in Scotland. It is the first study to provide an in-depth insight into the views of a range of people who are potentially interested in implementing the IM, an approach which is being delivered in more than 30 countries worldwide. As noted previously, there is surprisingly little scientific evidence regarding the IM published by those outside of the Planet Youth team (e.g. [30]). The study addresses this gap by providing important information for other areas considering the implementation of the IM using a relevant theoretical framework. Our study specifically engages with the challenge highlighted by Koning et al. (2020) by critically reviewing the evidence base and exploring cultural and contextual differences in relation to implementation [30]. The current study examined these cultural and contextual factors in relation to Scotland (with individual cities/areas also considered), related barriers and facilitators, and recommendations for policy, practice and research that are relevant to Scotland and beyond. Such strengths and weaknesses were also identified by Koning et al. [30]. Our study highlights the key considerations in implementing the IM in Scotland, which is essential in adapting interventions to new cultures and contexts [40, 41]. A recent qualitative study which examined the feasibility of implementing the IM in the US also highlighted the importance of cultural adaptation, as well as highlighting similar barriers to implementation including poverty/resources [42].

Using the CFIR [33] to illuminate our data allowed us to identify key components that are likely to be required when implementing the IM in Scotland. Our findings show that the strong evidence base for the IM provided a useful ‘selling point’ for the approach, with comparisons made between substance use in Iceland in the 1990s and in Scotland today. The desire for ‘something to be done’ in terms of primary, universal prevention strengthened participants’ view of the IM, as something that could, and potentially should, be implemented in Scotland to address rates of substance use and related harm. Data indicated that, if implemented, the IM would need to: be Scotland-specific; be adequately funded over the long-term; involve clear professional roles and responsibilities and leadership; involve a range of stakeholders, including young people; and have cross-party support locally and nationally. Participants were clear that, while current prevention work in Scotland is lacking, there are examples of good relevant services and work is therefore needed to foster collaborative partnership working between a range of organisations. Participants were clearly invested in substance use prevention, particularly when involving a multicomponent approach, but some were sceptical of the franchise model provided by Planet Youth and what would be provided for the investment. What is clear from our data is that participants viewed substance use prevention as requiring immediate investment and action.

### Strengths and limitations

This study generated rich description regarding implementation of a novel approach to primary prevention at a time when substance use harms in Scotland are at an all time high. The study was undertaken during the global COVID-19 pandemic. Despite the significant pressures on the services and individuals that were involved in this study, we managed to secure 16 interviews with a wide range of participants that illustrated a wide range of issues that should be taken account of in both considering whether to implement the IM and in consequent implementation should there be a decision to do so. Drawing on the CFIR during data analysis, rather than using it in the research design, means that some of the domains and constructs were not relevant. For example several constructs from three domains were not deemed relevant, nor was ‘outer setting’. Kirk et al. (2016) note that many studies using the CFIR often provide little detail of how the findings fit within the domains and constructs [34]. In this study we have provided clear detail regarding how the CFIR was used during data analysis and how our findings fit within each domain. Doing so has provided a greater theoretical understanding of our findings and considerable insight into the factors to consider for future implementation.

While we attempted to interview participants who were both ‘for’ and ‘against’ the IM, it was more difficult to find those who were ‘against’, likely due to our existing networks and being aware of those interested in the approach. This means that our findings likely reflect views of individuals who are more positive and knowledgeable about the IM. We did, however, engage with a range of participants across Scotland who had very different experiences and viewpoints of the IM, and primary prevention more generally. We were able to capture the views of those working in the field in third sector and statutory organisations, local and national government, and of family members, providing a wide range of views. Due to the challenges presented by COVID-19, and the inability to conduct interviews in person, we were unable to interview any young people so their voices are missing from the study. However, it is important to note that some young people in Scotland are supportive of exploring the model [43] and it would be important to involve young people in future studies.

### Implications for policy, practice and research

The study findings suggest that long term funding and strategic leadership is required to prevent substance use in Scotland. While rates of young people’s substance use in Scotland have been reducing in recent years [6], they are by no means low and drug and alcohol use appear to be increasing again [6]. Additionally, drug related deaths are increasing year on year, including increases among younger age groups [44], and are the highest in Europe [45]. This could mean implementing the IM or other evidence-based primary prevention approaches would be beneficial in addressing these issues. It is also important that a wide range of stakeholders are involved in the decision making and delivery of such activities to ensure they are appropriate for young people in different geographical areas. Relatedly, research is required to rigorously examine the impact of such interventions on young people’s substance use.

If the IM is to be implemented in Scotland, it is clear that it first needs to be piloted in a few areas to ensure it is transferable to the Scottish context and to start to develop an evidence base regarding its potential. The IM would need to be adjusted to ensure it is culturally appropriate for Scotland and fully funded to pay for both the survey (via Planet Youth) and the diversity of prevention activities that are required based on the surveys undertaken with young people. Building on current service provision is likely to be the most cost-effective and appropriate way of delivering such activities, as well as identifying gaps in provision and funding suitable services to address these gaps. In order to gain support and buy-in for the IM, it would be necessary to increase awareness of and knowledge about the approach to those who are likely to play a part including local, regional and national level decision makers and politicians, schools and educational organisations, communities and neighbourhoods, parents and carers, young people and their advocates, and a range of health and social care service providers both statutory and third sector. Digital and social media could play a key role in the dissemination of information, having the potential to reach a wide range of people. Identifying key organisations and individuals to lead the process would also be essential to ensure its success. Evaluation of pilot initiatives would be important to capture learning. Using a theory-based approach to data collection and analysis, such as the CFIR [33], would be beneficial to further elaborate on barriers and facilitators moving into implementation. Importantly, young people should be involved in research and evaluation concerning the IM and how it might make a difference to their experiences.

## Conclusions

This study provided insight into the views of a range of stakeholders regarding the potential implementation of the IM in Scotland, and perceived barriers and facilitators. The findings highlight the desire for higher prioritisation of universal primary prevention activities in Scotland, driven by concerns about high rates of substance use and related harms, and a general lack of effective and evidence based prevention activities across the country. There was support for the IM, as long as it was culturally appropriate and properly funded. Participants were clear that if the IM were introduced in Scotland, it would need to be piloted in several areas first. Wider dissemination of information about the IM and the need for prevention more generally would likely facilitate buy-in and support for the approach. If the IM is implemented, research and evaluation will be required to examine its potential and the outcomes of the approach.

## Supplementary Information

**Additional file 1.** Interview schedules

## Data Availability

The data-sets generated and/or analysed during the current study are not publicly available due to individual privacy potentially being compromised due to the small sample involved, but are available from the corresponding author on reasonable request.

## Abbreviations

ADP: Alcohol and Drug Partnership
CFIR: Consolidated Framework for Implementation Research
IM: Icelandic Model
MSP: Member of the Scottish Parliament
SALSUS: Scottish Schools Adolescent Lifestyle and Substance Use Survey

## References

[1] Bonomo Y, Proimos J (2005). ABC of adolescence: substance misuse: alcohol, tobacco, inhalants, and other drugs. Br Med J.

[2] Mirza KAH, Mirza S (2008). Adolescent substance misuse. Psychiatry.

[3] Howlett K, Williams T, Subramaniam G (2012). Understanding and treating adolescent substance abuse: a preliminary review. Focus (Madison).

[4] Degenhardt L, Stockings E, Patton G, Hall WD, Lynskey M (2016). The increasing global health priority of substance use in young people. Lancet Psychiatry.

[5] Currie C, Zanotti C, Morgan A, Currie D, de Looze M, Roberts C, et al. Social determinants of health and well-being among young people. Health Behaviour in School-aged Children (HBSC) Study: International report from the 2009/2010 survey [Internet]. Copenhagen: WHO Regional Office for Europe; 2012. Available from: http://www.euro.who.int/__data/assets/pdf_file/0003/163857/Social-determinants-of-health-and-well-being-among-young-people.pdf

[6] Scottish Government. Scottish schools adolescent lifestyle and substance use survey: National Overview (2018) [internet]. Edinburgh: Scottish Government; 2018. Available from: https://www.gov.scot/publications/scottish-schools-adolescent-lifestyle-substance-use-survey-salsus-national-overview-2018/

[7] ESPAD Group. ESPAD report 2019: results from the European school survey project on alcohol and other drugs [internet]. Luxembourg: European Monitoring Centre for Drugs and Drug Addiction; 2020. Available from: https://www.emcdda.europa.eu/system/files/publications/13398/2020.3878_EN_04.pdf

[8] Bremner P, Burnett J, Nunney F, Mistral W. Young people, alcohol and influences. A study of young people and their relationship with alcohol [Internet]. York: Joseph Rowntree Foundation; 2011. Available from: http://www.jrf.org.uk/publications/young-people-alcohol-and-influences

[9] Feinstein EC, Richter L, Foster SE (2012). Addressing the critical health problem of adolescent substance use through health care, research, and public policy. J Adolesc Health.

[10] European Monitoring Centre for Drugs and Drug Addiction (2019). European Prevention Curriculum: a handbook for decision-makers, opinion-makers and policy-makers in science-based prevention of substance use [Internet].

[11] Stockings E, Hall WD, Lynskey M, Morley KI, Reavley N, Strang J, Patton G, Degenhardt L (2016). Prevention, early intervention, harm reduction, and treatment of substance use in young people. Lancet Psychiatry.

[12] Faggiano F, Minozzi S, Versino E, Buscemi D (2014). Universal school-based prevention for illicit drug use [internet].

[13] Thomas R, McLellan J, Perera R. School-based programmes for preventing smoking. Cochrane Database Syst Rev. 2013;4. 10.1002/14651858.CD001293.pub3. 10.1002/14651858.CD001293.pub3 PMC7028068 23633306

[14] Foxcroft DR, Tsertsvadze A (2011). Universal family-based prevention programs for alcohol misuse in young people [internet].

[15] Gates S, McCambridge J, Smith L, Foxcroft D (2009). Interventions for prevention of drug use by young people delivered in non-school settings [internet].

[16] Stead M, Parkes T, Nicoll A, Wilson S, Burgess C, Eadie D, Fitzgerald N, McKell J, Reid G, Jepson R, McAteer J, Bauld L (2017). Delivery of alcohol brief interventions in community-based youth work settings: exploring feasibility and acceptability in a qualitative study. BMC Public Health.

[17] Scottish Government. Rights, respect and recovery [internet]. Edinburgh; 2018. Available from: https://www.gov.scot/publications/rights-respect-recovery/

[18] Cairney P, St Denny E (2020). Why isn’t government policy more preventive? [internet].

[19] Sigfúsdóttir ID, Kristjansson AL, Gudmundsdottir ML, Allegrante JP (2010). A collaborative community approach to adolescent substance misuse in Iceland. Int Psychiatry.

[20] Sigfusdottir ID, Thorlindsson T, Kristjansson AL, Roe KM, Allegrante JP (2008). Substance use prevention for adolescents: the Icelandic model. Health Promot Int [Internet].

[21] Kristjansson AL, Sigfusdottir ID, Thorlindsson T, Mann MJ, Sigfusson J, Allegrante JP (2016). Population trends in smoking, alcohol use and primary prevention variables among adolescents in Iceland, 1997-2014. Addiction.

[22] Kristjansson AL, James JE, Allegrante JP, Sigfusdottir ID, Helgason AR (2010). Adolescent substance use, parental monitoring, and leisure-time activities: 12-year outcomes of primary prevention in Iceland. Prev Med (Baltim) [Internet].

[23] Kristjansson AL, Mann MJ, Sigfusson J, Thorisdottir IE, Allegrante JP, Sigfusdottir ID (2020). Development and guiding principles of the Icelandic model for preventing adolescent substance use. Health Promot Pract.

[24] Kristjansson AL, Lilly CL, Thorisdottir IE, Allegrante JP, Mann MJ, Sigfusson J, Soriano HE, Sigfusdottir ID (2021). Testing risk and protective factor assumptions in the Icelandic model of adolescent substance use prevention. Health Educ Res.

[25] Sigfusdottir ID, Kristjansson AL, Gudmundsdottir ML, Allegrante JP (2011). Substance use prevention through school and community-based health promotion: a transdisciplinary approach from Iceland. Glob Health Promot.

[26] European Society for Prevention Research. The Icelandic model; is the hype justified? [Internet]. Palma; 2020. Available from: https://euspr.org/position-paper-of-the-european-society-for-prevention-research-on-the-icelandic-model/#:~:text=The Icelandic model is an,drug consumption among young people.

[27] Young E (2017). Iceland knows how to stop teen substance abuse but the rest of the world isn’t listening. The Independent [Internet].

[28] Milkman HB. Iceland Succeeds at Reversing Teenage Substance Abuse The U.S. Should Follow Suit [Internet]. Huffpost; 2017. Available from: https://www.huffpost.com/entry/iceland-succeeds-at-rever_b_9892758.

[29] Planet Youth. Planet Youth [Internet]. 2021 [cited 2021 May 12]. Available from: https://planetyouth.org/

[30] Koning IM, De Kock C, van der Kreeft P, Percy A, Sanchez ZM, Burkhart G (2020). Implementation of the Icelandic prevention model: a critical discussion of its worldwide transferability. Drugs Educ Prev Policy.

[31] Asgeirsdottir B, Kristjansson A, Sigfusson J, Allegrante J, Sigfusdottir I (2021). Trends in substance use and primary prevention variables among adolescents in Lithuania, 2006–19. Eur J Pub Health.

[32] Halsall T, Lachance L, Kristjansson AL (2020). Examining the implementation of the Icelandic model for primary prevention of substance use in a rural Canadian community: a study protocol. BMC Public Health.

[33] Damschroder LJ, Aron DC, Keith RE, Kirsh SR, Alexander JA, Lowery JC (2009). Fostering implementation of health services research findings into practice : a consolidated framework for advancing implementation science. Implement Sci.

[34] Kirk MA, Kelley C, Yankey N, Birken SA, Abadie B, Damschroder L (2016). A systematic review of the use of the Consolidated Framework for Implementation Research. Implement Sci.

[35] Scottish Government. Partnership working [Internet]. [cited 2021 May 18]. Available from: https://www.gov.scot/policies/alcohol-and-drugs/partnership-working/

[36] Maharaj N (2016). Using field notes to facilitate critical reflection. Reflective Pract.

[37] Ritchie J, Lewis J (2003). Qualitative research practice: a guide for social science students and researchers.

[38] Kiernan MD, Hill M (2018). Framework analysis: a whole paradigm approach. Qual Res J.

[39] Scotland E (2021). Building the curriculum [internet].

[40] Movsisyan A, Arnold L, Copeland L, Evans R, Littlecott H, Moore G (2021). Adapting evidence-informed population health interventions for new contexts: a scoping review of current practice. Heal Res Policy Syst.

[41] Schloemer T, Schröder-Bäck P (2018). Criteria for evaluating transferability of health interventions: a systematic review and thematic synthesis. Implement Sci.

[42] Kristjansson AL, Davis SM, Coffman J. Icelandic prevention model for rural youth: a feasibility study in central Appalachia. Health Promot Pract. 2021:1–10. 10.1177/15248399211002827. 10.1177/15248399211002827 33771042

[43] Weir K, Riggs P (2019). The road to recovery refresh: views from the Scottish youth parliament justice committee [internet]. Edinburgh.

[44] National Records of Scotland. Drug-related deaths in Scotland in 2019 [Internet]. Edinburgh; 2020 [cited 2021 May 12]. Available from: https://www.nrscotland.gov.uk/news/2020/drug-related-deaths-increase

[45] Nicholls J, Cramer S, Ryder S, Gold D, Priyadarshi S, Millar S, Hunter C, Hogg R, Jones A, Measham F, Stevens A, Hamilton I, McPhee I, Eastwood N, Powell M (2019). The UK government must help end Scotland’s drug-related death crisis. Lancet Psychiatry.

