# Supplementary material for: How might the ‘Icelandic model’ for preventing substance use among young people be developed and adapted for use in Scotland? Utilising the consolidated framework for implementation research in a qualitative exploratory study
Source: BMC Public Health. 2021 Sep 25;21:1742. doi: 10.1186/s12889-021-11828-z (PMC8464134; doi:10.1186/s12889-021-11828-z)
Supplement: Supplementary file 1 — Additional file 1. Interview schedules [file 12889_2021_11828_MOESM1_ESM.docx]

# Additional file 1

**Interview schedule – Dundee participants**

- What is your role/day to day job?
- What are your experiences of working with young people in relation to substance use?
- Do you think Dundee differs from other parts of Scotland in terms of young people’s substance use? If so, in what way?
- What activities/services are currently available to young people in Dundee as a way of preventing substance use?
- What services/activities do you think are lacking, if any?
- Who should play a role in preventing substance use among young people?
- What do you know about the Youth in Iceland/Icelandic model/Planet Youth approach?
- How did you hear about the approach?
- What do you think of the approach, in terms of:
  - The survey?
  - The activities that can be implemented?
    - Parenting activities
    - Sport/leisure
    - Schools
  - Any other aspects?
- Do you think the approach would work in Dundee? Why/why not? What might need to be adapted for a Dundee context?
- Do you think the approach would work in other areas of Scotland? Any in particular?
- If the approach was to implemented in Dundee and/or Scotland, what do you think would need to be taken into account in order to ensure it is successful?
- Is there anyone you think we should speak to as part of this project who might have different views to you?
- Is there anything else you would like to add?

**Interview schedule – Scotland participants**

- What is your role/day to day job?
- What are your experiences of working with young people in relation to substance use?
- What are your views on young people’s substance use in Scotland?
- What activities/services are currently available to young people in Scotland s a way of preventing substance use?
- What services/activities do you think are lacking, if any?
- Who should play a role in preventing substance use among young people?
- What do you know about the Youth in Iceland/Icelandic model/Planet Youth approach?
- How did you hear about the approach?
- What do you think of the approach, in terms of:
  - The survey?
  - The activities that can be implemented?
    - Parenting activities
    - Sport/leisure
    - Schools
  - Any other aspects?
- Do you think the approach would work in Scotland? Any in particular?
- If the approach was to be implemented in Scotland, what do you think would need to be taken into account in order to ensure it is successful?
- Is there anyone you think we should speak to as part of this project who might have different views to you?
- Is there anything else you would like to add?
